# Comparative Analysis of Volatile Compounds from Four Radish Microgreen Cultivars Based on Ultrasonic Cell Disruption and HS-SPME/GC–MS

**DOI:** 10.3390/ijms241914988

**Published:** 2023-10-08

**Authors:** Yuan Zhong, Zhilong Jia, Hailong Zhou, Dan Zhang, Guichen Li, Jihua Yu

**Affiliations:** 1 State Key Laboratory of Aridland Crop Science, Gansu Agricultural University, Lanzhou 730070, China; zhangdan@gsau.edu.cn (D.Z.); ligc@gsau.edu.cn (G.L.); 2 College of Science, Gansu Agricultural University, Lanzhou 730070, China; jiazl@gsau.edu.cn (Z.J.); 18894048561@163.com (H.Z.)

**Keywords:** flavor, volatile compounds, radish microgreens, ultrasonic cell disruption, GC–MS

## Abstract

The ultrasonic cell disruption method was used to efficiently extract isothiocyanates and other volatile compounds from radish microgreens. A total of 51 volatiles were identified and quantified by headspace solid-phase micro-extraction and gas chromatography–mass spectrometry (HS-SPME/GC–MS) in four radish microgreen cultivars, mainly including alcohols, aldehydes, isothiocyanates, sulfides, ketones, esters, terpenes, and hydrocarbons. The correlation between cultivars and volatile compounds was determined by chemometrics analysis, including principal component analysis (PCA) and hierarchical clustering heat maps. The aroma profiles were distinguished based on the odor activity value (OAV), odor contribution rate (OCR), and radar fingerprint chart (RFC) of volatile compounds. This study not only revealed the different flavor characteristics in four cultivars but also established a theoretical basis for the genetic improvement of radish microgreen flavors.

## 1. Introduction

Microgreens, an emerging category of edible cotyledonary leafy greens, are tender seedlings produced from the seeds of different species of vegetables, herbaceous plants, and aromatic herbs [1,2,3]. This category of potential functional foods is generally more flavorful and nutrient-dense than sprouts, baby greens, and mature vegetables [4,5]. In recent years, microgreens have gained increasing popularity as food ingredients by consumers because of their pleasing palette of colors, aromas, flavors, and textures [6,7].

Radish (*Raphanus sativus* L.), a root vegetable of the *Brassicaceae* family, is not only a common vegetable crop but also an important source of medicinal compounds [8]. Radish microgreens have a short production cycle of usually 5–10 days from seed germination to the edible stage [9,10]. To date, several studies have focused on nutrients and functions compared to their mature counterparts. Radish microgreens contain 2–4 times more ascorbic acid, 4.5 times more carotenoids, 4–5 times more isothiocyanates, and 976 times more α-tocopherol than mature vegetables [5]. They contain higher amounts of Mg, K, Ca, Se, P, and omega-3 fatty acids than those in mature vegetables [5]. However, to the best of our knowledge, there is no research on the flavor components and volatile compounds in radish microgreens.

Ultrasound technology has attracted considerable interest in food science and technology [11,12]. Bath sonicators provide indirect sonication, whereby the formation and distribution of cavities are nonuniform and weak, resulting in a longer metabolite extraction time [13]. By contrast, probe sonicators provide direct sonication, whereby the formed cavities, upon collapsing, result in an intensified vibration and uniform homogenous matrix, thereby reducing the extraction time and improving the overall efficiency of the process [14]. Ultrasound with a horn-type probe can result in efficient extraction and higher yields of metabolites in a shorter time compared to an ultrasound bath [15].

**Figure 1:**
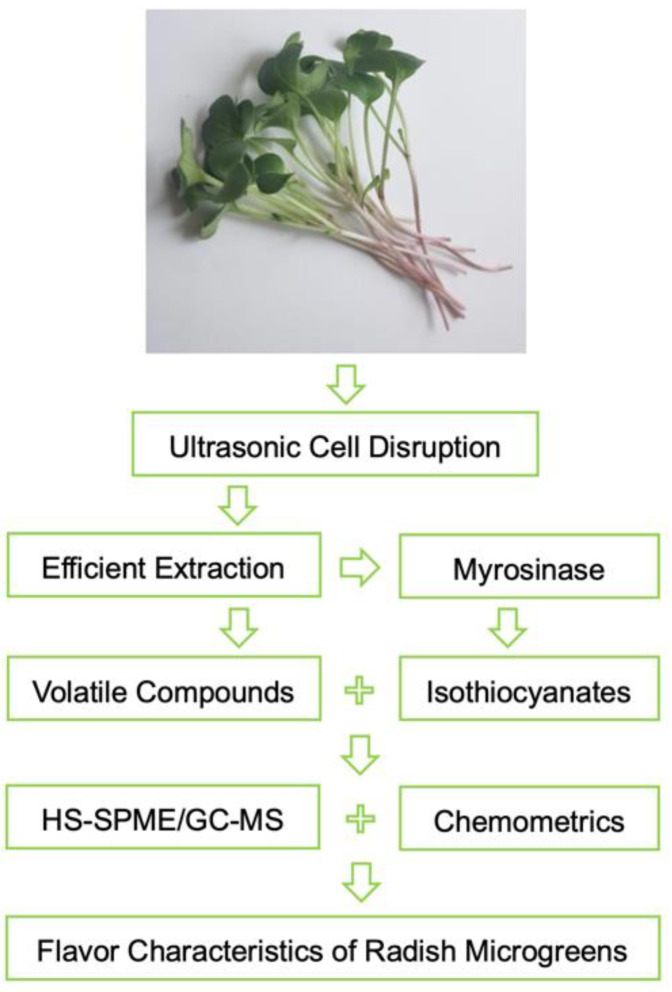
Flowchart for experimental design.

In this study, an ultrasonic cell disruption methodology was used to efficiently extract volatile compounds and accelerate the myrosinase-catalyzed breakdown of glucosinolates into bioactive isothiocyanates from radish microgreens (Figure 1). To understand the aromatic flavor characteristics and differences of volatile compositions, we determined the volatile compounds of four commercially available cultivars by using headspace solid-phase micro-extraction and gas chromatography–mass spectrometry (HS-SPME/GC–MS) combined with chemometrics analysis, including principal component analysis (PCA) and hierarchical clustering heat maps. Among the obtained volatiles, the odor activity value (OAV), odor contribution rate (OCR), and radar fingerprint chart (RFC) of volatile compounds were calculated to show the aroma profiles. Moreover, this study revealed that radish microgreens have more volatile compounds and aroma features compared to mature vegetables, as well as being rich in sulfides and glucosinolates. This work provides a theoretical basis for the eventual improvement of radish flavor and crop quality.

## 2. Results and Discussion

### 2.1. Detection of Volatile Compounds among Four Radish Microgreen Cultivars

A total of 51 different volatile compounds were detected in four radish microgreen cultivars by the HS-SPME/GC–MS methodology, including seven alcohols, ten aldehydes, thirteen isothiocyanates, four sulfides, two ketones, three esters, three terpenes, six hydrocarbons, and three miscellaneous compounds (Table 1). Of all these volatiles, the most abundant components were 4-methylthio-3-butenyl isothiocyanate (raphasatin), its trans- and cis-isomers (ranging from 5133.06 μg/kg in CRR to 13,968.25 μg/kg in HR), a breakdown product of glucodehydroerucin reported to be the main volatile constituent of radish roots responsible for their pungency [16,17,18,19]. Meanwhile, a high content of 4-(methylthio)butyl isothiocyanate (erucin) was also detected in all radish microgreen varieties. Therefore, the most salient characteristic was that most of the composition contained isothiocyanates, which endowed radish microgreens with a unique pungent odor and anti-cancer nutritional value. In addition, the volatile compounds 2-hexenal, nonanal, and decanal were the major aldehydes, and 6,10-dimethyl-5,9-undecadien-2-one was also well represented in ketones. The results showed that 25 kinds of volatiles were common to all varieties. The cultivars with the largest and lowest numbers of volatile compounds were HR (46 kinds) and WR (28 kinds), respectively. From a quantitative perspective, the total content of volatiles identified in the four cultivars was analyzed: 26,371.50 μg/kg in (HR), 13,801.56 μg/kg (CRR), 16,858.26 μg/kg (WR), and 13,959.40 μg/kg (CR). It could be concluded from the above analysis that the volatiles were greatly dependent on the cultivars.

### 2.2. Analysis of Volatile Compounds and Aroma Profile

**Figure 2:**
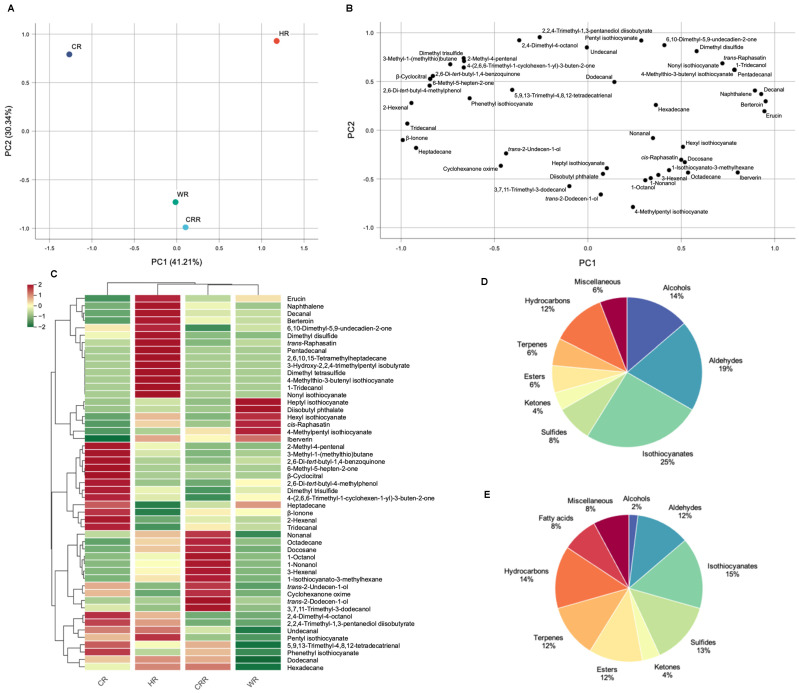
PCA score plot of four radish microgreen cultivars (**A**). The respective loading plot for PC1 and PC2 (**B**). Hierarchical clustering heat map analysis of volatile compounds (**C**). Classification and proportion of the 51 total volatile compounds detected in radish microgreens (**D**). Classification and proportion of the 51 total volatile compounds detected in the leaves and roots of radish (**E**).

Principal component analysis (PCA) was also an unsupervised clustering method requiring no prior knowledge of the dataset [20,21]. It could be observed from the two-dimensional PCA in Figure 2A,B that the four radish microgreen cultivars and their 51 volatile compounds formed corresponding groupings. The sum of the first two principal components reached 71.55%, of which PC1 represented 41.21% of the total variance, and PC2 represented 30.34% of the total variance, and the four radish microgreen varieties were divided into three clusters (Figure 2A). CR was located on the far-left side of PC1 (negative side), and HR exhibited both positive PC1 and PC2 score values. Two varieties, CRR and WR, were clustered tightly to the bottom side of the horizontal line representing PC2. Inspection of the corresponding loading plot in Figure 2B revealed that CR had higher contents of dimethyl trisulfide, 3-methyl-1-(methylthio)butane, 2-methyl-4-pentenal, and 4-(2,6,6-trimethyl-1-cyclohexen-1-yl)-3-buten-2-one, which accounted for its segregation. Moreover, HR showed higher contents of phenethyl isothiocyanate, 6,10-dimethyl-5,9-undecadien-2-one, dimethyl disulfide, trans-Raphasatin, and nonyl isothiocyanate, segregated towards the upper-right side of the score plot. WR and CRR displayed the highest contents of 4-methylpentyl isothiocyanate, segregated at the lower side of the loading plot.

The hierarchal clustering of the volatile compound profile of four radish microgreen cultivars was performed, and the results are shown in a heatmap (Figure 2C). According to the dendrogram, there were three clusters in the pattern of metabolite accumulation. These results were consistent with those derived from the above PCA analysis, indicating the significant differences in four radish microgreen cultivars. Future studies are needed to explore the molecular mechanisms regulating the variations of volatile compounds in different varieties.

Each category of volatiles was further compared with those in mature plants, as determined in a previous study [22]. As shown in Figure 2D, isothiocyanates had the highest contribution (approximately 25%), followed by aldehydes (19%) and alcohols (14%), and were the main components in radish microgreens in this study. These results are significantly different from those of the leaves and roots of radish in a previous study (Figure 2E), which may explain the special flavors of radish microgreens.

### 2.3. Odor Characteristics of Radish Microgreens Based on Odor Activity Value (OAV), Odor Contribution Rate (OCR), and Radar Fingerprint Chart (RFC)

**Figure 3:**
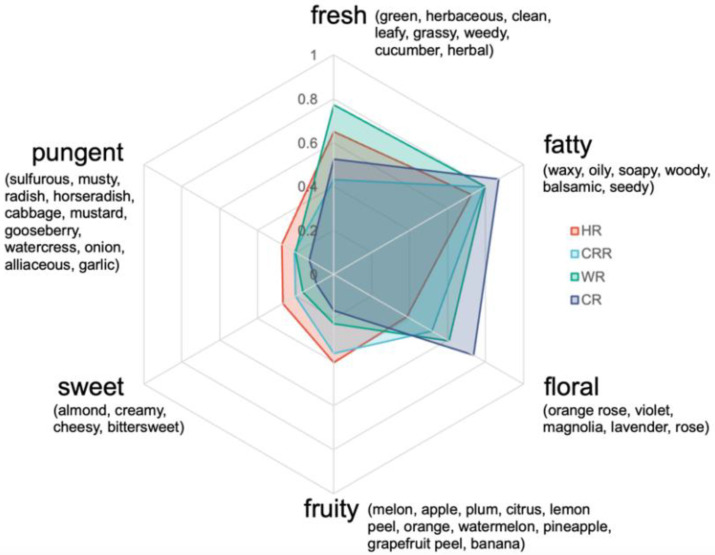
The aroma profiles of four radish microgreen cultivars.

The contribution of a volatile to the comprehensive flavor rested on the ratio of its actual concentration in the matrix to its odor threshold, which is known as the OAV [23]. As summarized in Table 2, the characteristic flavor of radish microgreens was approximately constituted by 24 important odorants. These 24 characteristic volatiles were divided into six aroma categories, including fresh, fatty, floral, fruity, sweet, and pungent. The radar fingerprint chart composed of them is depicted in Figure 3 [24]. The fatty odor was the strongest scent of radish microgreens, mainly including waxy, oily, soapy, woody, balsamic, and seedy aromas. All cultivars also possess an intensely fresh and floral odor, benefiting from aroma compounds 4-(2,6,6-Trimethyl-1-cyclohexen-1-yl)-3-buten-2-one and β-Ionone, which have extremely low odor threshold concentrations (0.007 μg/kg). Straight and longer chain aldehydes such as nonanal, decanal, and undecanal, oxidized from oleic acid in plants [25], had fresh, fatty, floral, and fruity characteristics and an especially high OCR in HR (6.2%, 26.7%, and 6.9%, respectively). Additionally, the odor descriptions of isothiocyanates (3-methylthiopropyl isothiocyanate, erucin, phenethyl isothiocyanate, and berteroin) and sulfides (dimethyl disulfide, dimethyl trisulfide, and dimethyl tetrasulfide) were roughly defined as a sulfurous aroma, including the aroma of horseradish, cabbage, mustard, gooseberry, watercress, onion, cabbage, alliaceous and garlic, all of which played an indispensable role in the pungent fragrance of radish microgreens.

### 2.4. Characteristic Volatile Isothiocyanates

**Figure 4:**
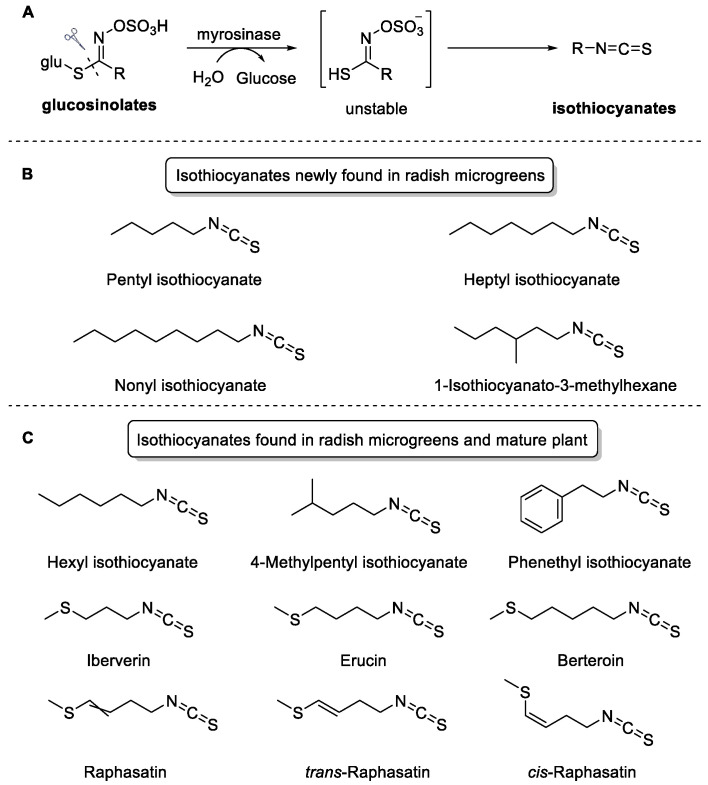
Structure of characteristic volatile isothiocyanates in radish microgreens.

Glucosinolates are hydrophilic and sulfur-containing plant secondary metabolites with over 130 variants, and they are particularly found in Brassica plants [32]. Although the primary function of glucosinolates in plants is not known, tissue disruption initiates a myrosinase-catalyzed breakdown, which yields glucose, unstable sulfate, and isothiocyanates (Figure 4A). Meanwhile, medical studies have highlighted the usefulness of combined glucosinolates and their breakdown products for supplementary health benefits, especially anti-inflammatory and antioxidant purposes. Four isothiocyanates, reported in some other plants (pentyl isothiocyanate in rocket leaves or kale, nonyl isothiocyanate in red sorrel, and heptyl isothiocyanate and 1-isothiocyanato-3-methylhexane in turnip) were newly found in radish microgreens (Figure 4B) [33,34,35,36]. Seven isothiocyanates were found in radish microgreens and mature plant tissue, such as sprouts, leaves, and roots (Figure 4C) [37,38,39].

## 3. Materials and Methods

### 3.1. Chemicals and Reagents

Ultrapure water was prepared by a Milli-Q ultrapure water machine (Millipore, Boston, MA, USA). The compound 2-octanol (Standard for GC, ≥99.5%) purchased from Aladdin Biochemical Technology Co., Ltd. (Shanghai, China) was used as an internal standard for quantitative analysis. Calcium chloride anhydrous (CaCl_2_) was supplied from Sinopharm Chemical Reagent Co., Ltd. (Shanghai, China).

### 3.2. Plant Materials

Four varieties of commercially available radish seeds were purchased from Cangzhou Jinke Lifeng Seedlings Co., Ltd., including Champion Radish (CR), Hailstone Radish (HR), China Rose Radish (CRR), and White Radish (WR). Radish microgreens were grown in an unheated greenhouse and under ambient light in Lanzhou, Gansu, China (36°03′ N, 103°73′ E). During the first 2 days, the trays were covered, and the seeds were germinated in the dark. For the next 7 days, the seedlings were exposed to light until harvesting.

### 3.3. Sample Preparation with Ultrasonic Cell Disruption Treatment

A total of 1.0 g of fresh radish microgreens was weighed, cut into pieces, and put into a 20 mL headspace reaction vial containing 4 mL of ultrapure water. Subsequently, 0.5 g of calcium chloride anhydrous was added to the vial. The sonication treatments were performed using a horn-type probe sonicator (6 mm diameter, 150 W, 20 kHz, JY96-IIN, Scientz, Ningbo, China) in a pulse mode. The parameters were set with the following conditions: ultrasound amplitude of 90%, durative time of 2 s, interval time of 2 s, total time of 5 min, and probe depth of 1 cm.

### 3.4. HS-SPME and GC–MS Analysis

After the ultrasonic cell disruption treatments, the internal standard 2-octanol (2 μL, 7.371 g/L in ethanol) was added. The mixture was placed with a magnetic stir bar and capped with a PTFE/silicone septum, followed by homogenization for 10 s in a water bath at 60 °C under stirring. The extraction and concentration of radish microgreen volatiles were performed by headspace solid-phase microextraction (HS-SPME) on the previous ultrasonicated extracts. Subsequently, HS-SPME extraction was carried out by exposing a 2-cm 50/30 μm DVB/CAR/PDMS fiber (Supelco, Bellefonte, PA, USA) to the headspace of the extracts for 40 min, at 60 °C, and under stirring. At the end of the extraction time, the fiber was immediately inserted into the GC split injection port for 5 min of thermal desorption, and the GC run was started. The same fiber was used for all the analyses.

The isolation and identification of volatile compounds were carried out using a gas chromatographer (GC-2030, Shimadzu, Kyoto, Japan) equipped with a mass spectrometry detector (GCMS-QP2020 NX, Shimadzu, Kyoto, Japan). The volatile compounds were separated on a DB-5 quartz capillary column (30 mm × 0.25 mm, 0.25 μm film thickness, Agilent Technologies, Santa Clara, CA, USA) with helium (≥99.999% purity) as the carrier gas at a flow rate of 1.0 mL/min. The split injection mode (split ratio = 5:1) was adopted during volatile insertion at 250 °C. The temperature program was initially set at 40 °C for 1 min and increased to 180 °C at a rate of 4 °C/min. It finally raised to 260 °C at 7 °C/min and was held for 3 min, with the entire procedure taking 50.43 min. The mass spectrometer was operated by the electron impact (EI) method with an ionization energy of 70 eV and a source temperature of 250 °C. Mass spectrometry uses the full-scan mode with a mass range from 35 *m*/*z* to 500 *m*/*z*. The filament current and quadrupole temperature were 150 μA and 250 °C, respectively.

### 3.5. Qualitative and Quantitative Analysis of Volatile Compounds

After the GC–MS analysis, every composition was analyzed by the computer workstation’s mass spectrometry library (NIST 17-1, NIST 17-2, and NIST 17s) according to its mass fragmentation pattern from the spectra database [40]. Only substances with an MS matching score greater than 75% were maintained. The concentration of each compound in the radish microgreens was calculated by the internal standard method, and the calculation formula was as follows: the content of each composition/(μg/kg) = (A1/A2) × (M1/M2) × 1000. A1 and A2 are the component areas of the detected composition and internal standard, respectively. M1 and M2 are the amounts of the internal standard and sample, respectively.

### 3.6. Statistical Analysis

All data were generated from three experiments, and analysis of variance was used to compare the volatile content of the radish microgreen cultivars. SPSS 26.0 software and Microsoft Excel 2019 were used for statistical analysis. The hierarchical clustering heat map was obtained using Metware Cloud, a free online platform for data analysis “https://cloud.metware.cn (accessed on 6 May 2023)”.

## 4. Conclusions

Aroma is an important characteristic of microgreens’ flavor. In this study, the ultrasonic cell disruption method was used to efficiently extract isothiocyanates and other volatile compounds from radish microgreens in a short time. A total of 51 types of volatile compounds were identified and quantified in four cultivars of radish microgreens by HS-SPME/GC–MS, mainly including alcohols, aldehydes, isothiocyanates, sulfides, ketones, esters, terpenes, and hydrocarbons. This study aimed to identify the correlation between cultivars and volatile compounds by chemometrics analysis, including PCA and heat maps. Among the obtained volatiles, the OAV and OCR of aroma compounds in the four cultivars of radish microgreens were calculated to reveal the aroma profiles. The fatty aroma was the strongest odor, followed by fresh and floral aromas. The evaluation of radish microgreen aroma composition helped to select varieties with special aroma characteristics and promote the breeding program. Moreover, four isothiocyanates were newly found in radish microgreens, and further investigations focused on the glucosinolates metabolic pathway of these characteristic isothiocyanates are warranted.

## Figures and Tables

**Table 1:** The composition and content of volatile compounds in four cultivars of radish microgreens by HS-SPME/GC–MS methodology.

No.	Compounds ^a^	CAS	RT ^b^ (min)	The Content of Volatile Compound in Different Cultivars ^c^ of Radish Microgreens (µg/kg) ^d^				Characteristic Ions (*m*/*z*) ^e^
				HR	CRR	WR	CR	
Alcohols								
1	1-Octanol	111-87-5	20.30	13.30	47.18	-	-	56, 70, 84, 112
2	*trans*-2-Dodecen-1-ol	69064-37-5	22.35	-	18.01	-	-	57, 82, 95, 109, 138, 166, 184
3	1-Nonanol	143-08-8	23.85	25.04	79.18	-	-	56, 70, 83, 126
4	*trans*-2-Undecen-1-ol	75039-84-8	26.97	17.90	26.01	16.79	23.09	57, 68, 82, 109, 152, 170
5	3,7,11-Trimethyl-3-dodecanol	7278-65-1	29.94	24.78	232.04	22.02	65.10	43, 73, 83, 111, 139, 199, 213
6	2,4-Dimethyl-4-octanol	33933-79-8	34.54	19.36	-	-	31.48	57, 59, 101, 111, 143
7	1-Tridecanol	112-70-9	36.11	14.03	-	-	-	55, 69, 83, 111, 139, 154, 182
Aldehydes								
8	2-Methyl-4-pentenal	5187-71-3	9.19	14.26	-	-	51.77	41, 69, 83
9	3-Hexenal	4440-65-7	9.73	16.82	46.03	-	-	41, 69, 98
10	2-Hexenal	505-57-7	11.96	160.91	374.90	299.24	897.95	41, 69, 83
11	Nonanal	124-19-6	21.58	453.96	548.23	269.86	365.36	57, 70, 98, 114, 142
12	Decanal	112-31-2	25.30	490.84	322.46	311.43	256.83	57, 70, 83, 112, 138, 156
13	Undecanal	112-44-7	28.84	47.44	33.60	19.62	46.80	57, 82, 96, 126, 142
14	Tridecanal	10486-19-8	31.54	20.27	26.23	23.85	32.34	57, 68, 82, 110, 154, 180
15	Dodecanal	112-54-9	32.16	35.76	32.85	-	30.34	41, 67, 82, 110, 140, 156, 184
16	Pentadecanal	2765-11-9	33.70	10.91	-	-	-	57, 68, 82, 110, 138, 154, 182, 208
17	5,9,13-Trimethyl-4,8,12-tetradecatrienal	66408-55-7	42.24	22.97	32.16	-	40.87	41, 69, 93, 124, 136, 161, 179, 205, 248
Isothiocyanates								
18	Pentyl isothiocyanate	629-12-9	21.31	37.45	7.46	-	24.04	43, 72, 101, 129, 131
19	4-Methylpentyl isothiocyanate	17608-07-0	23.69	169.02	202.84	263.39	140.52	43, 72, 101, 128, 143
20	Nonyl isothiocyanate	4430-43-7	23.78	15.17	-	-	-	41, 72, 96, 115, 152, 156, 184
21	Hexyl isothiocyanate	4404-45-9	25.06	399.11	248.27	508.09	224.60	43, 72, 100, 115, 143
22	4-Methylthio-3-butenyl isothiocyanate (Raphasatin)	51598-96-0	25.73	12.97	-	-	-	45, 72, 87, 112, 159
23	Heptyl isothiocyanate	4426-83-9	27.31	44.33	36.77	82.31	38.10	43, 72, 100, 124, 142, 157
24	1-Isothiocyanato-3-methylhexane	206761-72-0	27.60	6.22	14.23	-	-	43, 72, 100, 114, 142, 156
25	3-Methylthiopropyl isothiocyanate (Iberverin)	505-79-3	29.08	130.44	98.71	151.84	-	41, 72, 101, 147
26	(*Z*)-4-Methylthio-3-butenyl isothiocyanate (*cis*-Raphasatin)	123954-93-8	32.54	2390.87	1959.88	2911.14	1736.70	45, 72, 87, 112, 159
27	4-(Methylthio)butyl isothiocyanate (Erucin)	4430-36-8	33.05	8212.93	5049.85	6142.74	3741.82	55, 61, 85, 115, 146, 161
28	(*E*)-4-Methylthio-3-butenyl isothiocyanate (*trans*-Raphasatin)	13028-50-7	33.14	11,564.41	3173.18	4483.32	4453.34	45, 72, 87, 112, 142, 159
29	Phenethyl isothiocyanate	2257-09-2	33.96	12.00	25.17	-	37.61	51, 65, 91, 128, 135, 163
30	1-Isothiocyanato-5-(methylthio)pentane (Berteroin)	4430-42-6	36.54	502.58	218.24	174.79	57.01	41, 61, 101, 129, 142, 175, 178
Sulfides								
31	Dimethyl disulfide	624-92-0	8.48	38.44	-	-	10.70	45, 79, 94
32	Dimethyl trisulfide	3658-80-8	16.58	16.10	14.10	15.96	19.40	45, 79, 82, 126, 130
33	3-Methyl-1-(methylthio)butane	13286-90-3	22.71	62.74	34.73	48.23	159.19	55, 70, 103, 118
34	Dimethyl tetrasulfide	5756-24-1	26.49	8.32	-	-	-	45, 79, 94, 111, 143, 158
Ketones								
35	6-Methyl-5-hepten-2-one	110-93-0	16.18	-	-	-	20.35	43, 69, 83, 108
36	6,10-Dimethyl-5,9-undecadien-2-one	689-67-8	33.35	576.27	206.14	333.40	399.23	43, 69, 93, 107, 136, 161, 194
Esters								
37	3-Hydroxy-2,2,4-trimethylpentyl isobutyrate	77-68-9	31.07	58.03	-	-	-	56, 71, 89, 113, 143, 173
38	2,2,4-Trimethyl-1,3-pentanediol diisobutyrate	6864-50-0	37.66	37.72	27.42	26.76	41.53	43, 71, 83, 111, 143, 159, 185, 215, 243
39	Diisobutyl phthalate	84-69-5	44.30	35.34	25.09	135.12	23.05	57, 76, 104, 121, 149, 167, 195, 223, 278
Terpenes								
40	β-Cyclocitral	432-25-7	24.29	-	-	-	23.98	41, 67, 91, 123, 137, 154
41	4-(2,6,6-Trimethyl-1-cyclohexen-1-yl)-3-buten-2-one	14901-07-6	33.55	50.34	17.82	58.62	102.52	43, 77, 91, 121, 135, 177, 192
42	β-Ionone	79-77-6	33.68	-	41.73	37.93	75.42	43, 77, 91, 107, 135, 177, 192
Hydrocarbons								
43	Naphthalene	91-20-3	24.93	149.27	105.57	92.73	86.23	51, 64, 102, 128
44	Hexadecane	544-76-3	38.37	38.98	40.48	-	23.34	57, 71, 85, 113, 141, 155, 183, 226
45	Heptadecane	629-78-7	40.45	-	13.15	25.59	30.64	57, 71, 85, 113, 141, 169, 183, 211, 240
46	2,6,10,15-Tetramethylheptadecane	54833-48-6	40.94	44.29	-	-	-	57, 71, 85, 113, 141, 155, 183, 211, 239, 267, 296
47	Octadecane	593-45-3	42.75	57.23	78.92	38.57	32.75	57, 71, 85, 113, 141, 155, 183, 211, 226, 254
48	Docosane	629-97-0	43.09	43.02	77.93	-	-	57, 71, 85, 113, 141, 155, 183, 211, 239, 253, 281, 310
Miscellaneous								
49	Cyclohexanone oxime	100-64-1	24.81	20.99	58.73	22.30	44.17	41, 59, 98, 113
50	2,6-Di-*tert*-butyl-1,4-benzoquinone	719-22-2	33.93	15.43	12.70	16.91	39.41	41, 67, 91, 121, 135, 177, 178, 220
51	2,6-Di-*tert*-butyl-4-methylphenol	128-37-0	35.16	232.94	193.57	325.71	531.82	57, 81, 105, 119, 145, 177, 189, 205
The total number of volatile compounds				46	39	28	37	
The total content of volatile compounds (μg/kg)				26,371.50	13,801.56	16,858.26	13,959.40	

^a^ Volatile compounds were integrated with the GC–MS automatic deconvolution system and compared with the standard mass spectrum in the mass spectrometry library (NIST 17-1, NIST 17-2, and NIST 17s). All category volatile compounds are listed in order of retention time. ^b^ RT: retention time. ^c^ HR: Hailstone Radish; CRR: China Rose Radish; WR: White Radish; CR: Champion Radish. ^d^ Each value is the mean of triplicate biological samples taken from the same radish microgreen cultivar; “-”, not detected. ^e^ The characteristic ion (*m*/*z*) was employed for selecting the corresponding compound to avoid possible interference by other volatiles.

**Table 2:** OAV and OCR of the 24 most potent volatile compounds in the four radish microgreen cultivars.

Group (No.) ^a^	Volatile Compound	CAS	OTS ^b^ μg/kg	HR		CRR		WR		CR		Flavor Description ^e^
				OAV ^c^	OCR ^d^	OAV	OCR	OAV	OCR	OAV	OCR	
1	1-Octanol	111-87-5	42	0.32	0.001	1.12	0.006	-	0	-	0	green, herbaceous, waxy, oily, sweet
3	1-Nonanol	143-08-8	34	0.74	0.003	2.33	0.012	-	0	-	0	green, clean, oily, orange rose
9	3-Hexenal	4440-65-7	0.12	140.17	0.609	383.58	1.899	-	0	-	0	leafy, grassy, weedy, melon, apple, fatty
10	2-Hexenal	505-57-7	17	9.47	0.041	22.05	0.109	17.60	0.073	52.82	0.146	sweet, almond, apple, plum, green, leafy
11	Nonanal	124-19-6	0.32	1418.63	6.163	1713.22	8.480	843.31	3.508	1141.75	3.147	waxy, orange rose, citrus, green, lemon peel, cucumber
12	Decanal	112-31-2	0.08	6135.50	26.654	4030.75	19.952	3892.88	16.193	3210.38	8.850	sweet, citrus, orange, waxy, green
13	Undecanal	112-44-7	0.03	1581.33	6.870	1120.00	5.544	654.00	2.720	1560.00	4.300	soapy, waxy, watermelon, pineapple
14	Tridecanal	10486-19-8	8	2.53	0.011	3.28	0.016	2.98	0.012	4.04	0.011	clean, soapy, waxy, citrus, grapefruit peel
15	Dodecanal	112-54-9	0.13	275.08	1.195	252.69	1.251	-	0	233.38	0.643	soapy, waxy, woody, violet
16	Pentadecanal	2765-11-9	430	<1	0	-	0	-	0	-	0	fresh, waxy
25	3-Methylthiopropyl isothiocyanate	505-79-3	5	26.09	0.113	19.74	0.098	30.37	0.126	-	0	sulfurous, radish, horseradish, cabbage, mustard
27	4-(Methylthio)butyl isothiocyanate (Erucin)	4430-36-8	3	2737.64	11.893	1683.28	8.332	2047.58	8.517	1247.27	3.438	cabbage, radish
29	Phenethyl isothiocyanate	2257-09-2	6	2.00	0.009	4.20	0.021	-	0	6.27	0.017	horseradish, gooseberry, watercress
30	1-Isothiocyanato-5-(methylthio)pentane (Berteroin)	4430-42-6	800	<1	0	<1	0	<1	0	<1	0	cabbage, radish
31	Dimethyl disulfide	624-92-0	0.16	240.25	1.044	-	0	-	0	66.88	0.184	sulfurous, onion, cabbage
32	Dimethyl trisulfide	3658-80-8	0.006	2683.33	11.657	2350.00	11.632	2660.00	11.065	3233.33	8.913	sulfurous, alliaceous, onion
34	Dimethyl tetrasulfide	5756-24-1	0.02	416.00	1.807	-	0	-	0	-	0	garlic, sulfurous
35	6-Methyl-5-hepten-2-one	110-93-0	50	-	0	-	0	-	0	<1	0	apple, creamy, cheesy, banana, bittersweet
36	6,10-Dimethyl-5,9-undecadien-2-one	689-67-8	60	9.60	0.042	3.44	0.017	5.56	0.023	6.65	0.018	magnolia, lavender, rose, leafy, green, apple, banana
40	β-Cyclocitral	432-25-7	3	-	0	-	0	-	0	7.99	0.022	green, herbal, sweet
41	4-(2,6,6-Trimethyl-1-cyclohexen-1-yl)-3-buten-2-one	14901-07-6	0.007	7191.43	31.241	2545.71	12.601	8374.29	34.835	14,645.71	40.372	violet, woody, green
42	β-Ionone	79-77-6	0.007	-	0	5961.43	29.509	5418.57	22.540	10,774.29	29.700	balsamic, rose, violet, woody, seedy
43	Naphthalene	91-20-3	1	149.27	0.648	105.57	0.523	92.73	0.386	86.23	0.238	pungent
51	2,6-Di-*tert*-butyl-4-methylphenol	128-37-0	1000	<1	0	<1	0	<1	0	<1	0	musty

^a^ The serial numbers of the volatile compounds are consistent with Table 1. ^b^ OTS: odor threshold. The OTS of volatile compounds was obtained from the following report [26,27,28,29,30,31]. ^c^ OAV: odor activity value, OAV = odorant concentration/odorant threshold, “-”, not detected. ^d^ OCR: odor contribution rate, OCR (%) = the OAV of each odorant/total OAVs of all odorants. ^e^ Flavor description, respectively, obtained from the online database “http://www.thegoodscentscompany.com (accessed on 20 April 2023)”.

## Data Availability

Not applicable.
